# Retraction: The Development of Illness Anxiety Disorder in a Patient After Partial Thyroidectomy

**DOI:** 10.7759/cureus.r59

**Published:** 2022-06-14

**Authors:** Neeraj Kancherla, Srija Chowdary Vanka, Sandesh Pokhrel, Reshma Bano Shahzadi, Ganipineni Vijaya Durga Pradeep

**Affiliations:** 1 Psychiatry, King George Hospital, Visakhapatnam, IND; 2 Psychiatry, Dr. Pinnamaneni Siddhartha Institute of Medical Sciences and Research Foundation, Chinna Avutapalle, IND; 3 Psychiatry, Nepal Medical College, Lalitpur, NPL; 4 Psychiatry, N.K.P. Salve Institute of Medical Sciences and Research Center, Nagpur, IND; 5 General Medicine, SRM Medical College Hospital & Research Center, Chennai, IND; 6 General Medicine, King George Hospital, Visakhapatnam, IND

This article has been retracted due to the unauthorized submission and publication of this case by Neeraj Kancherla. Shortly after publication of this case report, the Cureus editorial office was contacted by Dr. Aqeel Hashmi (Medical Director at Westpark Springs Hospital, Richmond, Texas) with the allegation that Mr. Kancherla had fraudulently published this case without the knowledge or consent of Dr. Hashmi, who was the primary physician in this case originating from his private practice.

Dr. Hashmi stated the following: "The first author Neeraj Kancherla recently rotated with me at Westpark Springs Hospital in Richmond, Texas USA. I can verify that the case mentioned is from my private practice and I have emails and medical records to show communication with Neeraj. I can also verify that I did not give consent for this work to be submitted."

When confronted with these allegations, Mr. Kancherla admitted to taking the data and writing and submitting the case report without alerting Dr. Hashmi. Mr. Kancherla's co-authors all claim to have been unaware of the fraudulent origin of the case data. After reviewing the aforementioned documents and contacting both Dr. Hashmi and Mr. Kancherla, Cureus has made the decision to formally retract the article.

NOTE: This retraction notice has been updated on June 15th, 2022 to more accurately describe the reason for retraction: "unauthorized submission and publication of this case."

